# Public/community engagement in health research with men who have sex with men in sub-Saharan Africa: challenges and opportunities

**DOI:** 10.1186/s12961-016-0106-3

**Published:** 2016-05-27

**Authors:** Sassy Molyneux, Salla Sariola, Dan Allman, Maartje Dijkstra, Evans Gichuru, Susan Graham, Dorcas Kamuya, Gloria Gakii, Brian Kayemba, Bernadette Kombo, Allan Maleche, Jessie Mbwambo, Vicki Marsh, Murugi Micheni, Noni Mumba, Michael Parker, Jasmine Shio, Clarence Yah, Elise van der Elst, Eduard Sanders

**Affiliations:** Department of Health Systems and Research Ethics, KEMRI/Wellcome Trust Research Programme (KWTRP), Kilifi, Kenya; The Ethox Centre, Department of Public Health, University of Oxford, Oxford, UK; The Centre for Clinical Vaccinology and Tropical Medicine, Nuffield Department of Medicine, University of Oxford, Oxford, UK; Faculty of Social Sciences, University of Torku, Torku, Finland; Dalla Lana School of Public Health, University of Toronto, Toronto, Canada; Department of Global Health, Academic Medical Centre, University of Amsterdam, Amsterdam, The Netherlands; Kenya Research Group, University of Washington, Seattle, USA; University of Nairobi, Nairobi, Kenya; University of Manitoba, Manitoba, USA; University of Cape Town, Cape Town, South Africa; KELIN – Reclaiming rights, Rebuilding Live, Nairobi, Kenya; Department of Psychiatry and Mental Health, Muhimbili University of Health and Allied Sciences, Dar es Salaam, Tanzania; Department of Project Management, Deloitte Consulting Ltd, Dar es Salaam, Tanzania; Wits Reproductive Health & HIV Institute, Faculty of Health Sciences, University of the Witwatersrand, Johannesburg, South Africa

## Abstract

**Background:**

Community engagement, incorporating elements of the broader concepts of public and stakeholder engagement, is increasingly promoted globally, including for health research conducted in developing countries. In sub-Saharan Africa, community engagement needs and challenges are arguably intensified for studies involving gay, bisexual and other men who have sex with men, where male same-sex sexual interactions are often highly stigmatised and even illegal. This paper contextualises, describes and interprets the discussions and outcomes of an international meeting held at the Kenya Medical Research Institute-Wellcome Trust in Kilifi, Kenya, in November 2013, to critically examine the experiences with community engagement for studies involving men who have sex with men.

**Discussion:**

We discuss the ethically charged nature of the language used for men who have sex with men, and of working with ‘representatives’ of these communities, as well as the complementarity and tensions between a broadly public health approach to community engagement, and a more rights based approach. We highlight the importance of researchers carefully considering which communities to engage with, and the goals, activities, and indicators of success and potential challenges for each. We suggest that, given the unintended harms that can emerge from community engagement (including through labelling, breaches in confidentiality, increased visibility and stigma, and threats to safety), representatives of same-sex populations should be consulted from the earliest possible stage, and that engagement activities should be continuously revised in response to unfolding realities. Engagement should also include less vocal and visible men who have sex with men, and members of other communities with influence on the research, and on research participants and their families and friends. Broader ethics support, advice and research into studies involving men who have sex with men is needed to ensure that ethical challenges – including but not limited to those related to community engagement – are identified and addressed.

**Summary:**

Underlying challenges and dilemmas linked to stigma and discrimination of men who have sex with men in Africa raise special responsibilities for researchers. Community engagement is an important way of identifying responses to these challenges and responsibilities but itself presents important ethical challenges.

## Background

Community engagement (CE) is increasingly promoted globally, including for health research conducted in developing countries [1]. CE is promoted for diverse reasons, ranging from the potential to increase community participation, through the ability to improve the design and implementation of studies, to the desire to transform what can be inequitable power relations between researchers and participants. CE is also promoted for its intrinsic importance; as a way of ensuring that communities are shown appropriate levels of respect. Despite this growing attention, the meaning of the term ‘community engagement’, what is expected and to what ends and how it is implemented and evaluated in practice, remain varied, unclear, contested and under-researched [2–6]. One illustration is the fuzzy distinction between public engagement and CE. We follow others in using the term CE quite broadly, to suggest engagement with a diverse range of communities or individuals potentially involved in the research, either directly as participants or indirectly as fellow community members or residents in areas where research might take place [7]. Thus, the general public in locations where research is being planned or conducted are potentially key communities to include in CE initiatives. A particular challenge of CE identified in recent literature has been that researchers’ goals for CE are often implied rather than clearly articulated [1]. This is an important limitation because many CE initiatives and activities have diverse goals, which may be incongruous with one another. Furthermore, all CE has the potential to have a negative impact, at the very least through taking up people’s time, but also through unintended outcomes such as making some individuals feel obliged to take part in research, or by raising expectations that cannot be met [1, 8–11].

In sub-Saharan Africa, CE needs and challenges are arguably intensified for studies involving men who have sex with men (MSM) [12]. Male same-sex sexual behaviours are not only highly stigmatised in many settings, but may also be illegal [13]. The need for appropriate health research with MSM is underscored in this context. Research conducted among MSM in the region has revealed that many MSM contend with overlapping health and social needs, and significant information gaps [14–21]. MSM, for example, are at high risk of acquiring or transmitting HIV-1 and other sexually transmitted diseases, and many face barriers to care as a result of structural and social inequality that lead to stigma, discrimination and social isolation. In these settings, studies that produce new knowledge with the potential to positively impact on inclusion, treatment and support of MSM are clearly much needed, but require careful design and implementation, including with regards to CE.

There are a number of papers and guidelines suggesting appropriate approaches to engage with communities for health research in general, and for HIV studies and research involving MSM more specifically [3, 6, 22–26]. Notable for research involving MSM in sub-Saharan Africa is the document ‘Respect, protect and fulfil: best practices guidance in conducting HIV research with gay, bisexual and other MSM in rights constrained environments’ [27]. This document is invaluable in assisting researchers and community organisations to better design and conduct meaningful HIV and other research involving MSM in providing a checklist of factors for researchers and community organisations to consider in studies, and in offering lessons learned through a series of case studies. Like other CE literature and guidance, the document highlights the complexity of designing and implementing CE strategies, and illustrates the importance of approaches that are tailored to specific studies in particular contexts. The document also emphasises the need to rethink and amend CE approaches and activities in response to emerging issues and needs.

While such guidance exists (see also http://www.fhi360.org/resource/stakeholder-engagement-toolkit-hiv-prevention-trials), as well as an interactive toolkit to monitor and evaluate the impact of CE work [22, 28], it was felt that discussion about the successes and challenges of conducting CE in what are often ethically and politically fraught contexts continues to be needed. At the Kenya Medical Research Institute (KEMRI)-Wellcome Trust research programme in Kilifi, research conducted for over 10 years among MSM has in some instances faced substantial challenges [29]. Not least was a physical attack on the KEMRI research clinic in peri-urban Mtwapa, where MSM study participants were specifically targeted. In response to this event – and through discussion with diverse community representatives – CE goals and activities for studies conducted by the programme involving MSM were revised and revamped, including through regular planning and feedback meetings between researchers, CE facilitators employed by the KEMRI-Wellcome Trust Programme, and diverse community representatives. Given the continuing underlying challenges and dilemmas linked to stigma and discrimination, and recognition of the need to continuously reflect upon appropriate approaches for CE for studies involving MSM, several authors of this paper (see author attributions at the end of the paper) organized a meeting to share CE lessons and challenges with others conducting research with MSM in sub-Saharan Africa. This paper discusses the key challenges and issues raised in that meeting. We suggest that, while CE is often presented as supporting ethical research practice, as a way of mitigating harms of research, and even as emancipatory, CE itself can also be harmful. We suggest various strategies for engagement and approaches to minimise such harms.

## Workshop organization and participants

A total of 26 individuals from 11 institutions attended a one and a half day meeting in Kilifi, Kenya, in 2013. The focus of the meeting was on CE in health research involving MSM in sub-Saharan Africa, and the majority of invitees had such experience, either as researchers or as community liaison personnel. In addition to participants from seven different institutions in South and East Africa involved in MSM research, we invited advocates and ethicists with experience of working with MSM in the region to enrich the discussions. Although participants were not asked to share their sexual orientation at the meeting, it was clear throughout discussions that a range of sexual orientations were represented in the group.

Workshop discussions were based on questions and dilemmas raised regularly in CE meetings at the KEMRI-Wellcome Trust programme in Kilifi. They were organized around the following themes:

- What is ultimately the goal (or range of goals) of engaging communities in research with MSM in contexts like ours? How do these goals differ depending on different interests of key actors, to what effect and for whom?
- How might the range of communities to be engaged with be identified? Who represents these communities and how, and through which forms of engagement can this representation be facilitated? What should be done when representatives of different communities have opposing views of what should be done or how activities might be taken forwards?
- What are the responsibilities of researchers with regards to tackling the health needs and underlying factors contributing to health problems, stigma and discrimination, and where do those responsibilities end?
- What kinds of networks and collaborations might play a role in translating research findings into evidence-based policies and programmes? What types of activities can facilitate this ‘research uptake’ or ‘knowledge transfer’?

The questions outlined above were discussed in plenary sessions and in small group discussions that were tape recorded. At the end of the first day, organisers of the meeting drew upon notes from all of the discussions to identify the range of issues identified. This overview was fed back to all participants at the end of the meeting to share our interpretation and elicit further discussion. In this session, participants commented that it would be valuable to share the discussions more widely, including through scientific publication.

Given the complex nature of many of the topics raised and discussed, we recognised that we would not be able to reach conclusions by the end of the meeting. However, of interest to us all were the many convergences and differences among meeting participants in general approaches to CE for studies involving MSM. Given the ethical nature of some of these overlaps, debates and differences, these are valuable to a wider audience as ‘food for thought’ or as ideas and issues to consider in planning for engagement and research involving MSM. The paper also highlights areas for further discussion and research, and re-emphasises the importance of including representatives of MSM populations in the development of CE plans and activities from the earliest possible stage in all contexts.

## Key discussion points and emerging issues

### Language is ethically charged

From the outset of the meeting, it was clear that the language used in discussing male-to-male sex has social meaning and ramifications. We adopted the term MSM to capture the full range of male-to-male sexual contact given that many of the biomedical and social scientific studies we work on focus on reducing risks of HIV and improving access to quality services. This behavioural definition used in epidemiological studies was first used in the 1990s with the deliberate aim of moving away from sexual orientation or identity categories (homosexual, bisexual, heterosexual, or gay, bi and straight) in order to recognise and advocate for improved services for all men engaging in same-sex sexual behaviours, regardless of orientation or identity [13]. However, as noted in the amfAR guidelines [27], the term MSM can cover a broad and diverse group of individuals, some of whom would more readily self-identify under another umbrella term such as ‘lesbian, gay, bisexual, and transgender’ (LGBT), or Gay-Other MSM-Transgender (GMT), or who may identify with a particular sub-community or with no umbrella or particular sub-community at all.

At the meeting, it was recognised that using the term MSM can be seen as a failure to recognise and embrace individual identities, as attempting to depoliticise the issues faced, and as failing to recognise and strive for equity and civil and human rights for sexual and gender minorities. It can also be seen as potentially masking what may be different interests, needs or agendas of, for example, sex workers, gay men, and transgender individuals, including in relation to healthcare access and equity. Further problems of using any term are that many men are not open about their behaviour or identity in many contexts, and that any labelling has the potential to increase stigma and segregation. Thus, it should be recognised that any terms used are ethically charged, can be shaped by, connected to, and feed into deep-rooted structural injustices and exclusion, and can have potentially important implications for all aspects of CE for studies involving MSM.

### Public health or human rights approach to community engagement?

In our meeting, two broad approaches to CE emerged in discussions: (1) the perspectives of researchers, reviewers, and funders regarding general expectations of socially responsible research and ethics in contexts of shortages in multiple basic needs and resources, and major local and global inequities (a predominantly public health approach); and (2) the perspective of advocates concerned about the very specific vulnerabilities and challenges faced by study participants – homophobia, stigma, marginalisation and hostile environments – with a more politicised approach to HIV research (a predominantly rights-based approach).

Although these two different perspectives could and do contribute to some tension with regards to perceptions of appropriate forms of CE, there were also significant overlaps in values and principles. There were three main areas in which differences or debates, and ultimately overlaps, were observed.

Firstly, there were differences in discussions about whether the goals for CE are generally instrumental (for science) or intrinsic. Instrumental goals might, for example, be to identify what the ‘right’ study question or approach should be, how the research might be done most appropriately for/with MSM communities, what benefits are appropriate or how findings are most usefully publicised. Intrinsic goals might be in order to ensure respect, and because there is simply no other way that research can appropriately be done, as captured by the term ‘nothing about us without us’. In practice, however, and also observed in CE more generally, it became clear that these two sets of goals are not always easily distinguished, and that researchers often have multiple, not necessarily clearly articulated, and in some cases conflicting goals. Different goals may also be articulated in different contexts for different audiences, depending on what is felt to be of greatest relevance or acceptability. For example, a goal to work with MSM representatives to identify appropriate levels of benefits and to minimise risks for all study participants might be emphasised in communication with ethics committees, whereas to LGBT groups, a goal to ensure equitable access to healthcare regardless of sexuality could be more centrally discussed. While improved recruitment would be an outcome for researchers, benefit such as access to care for MSM as well as wider communities would be an important, intrinsic value in itself.

A second area of difference and debate was whether researchers should engage in open advocacy, or whether advocacy efforts on behalf of MSM communities should be more ‘under the radar’. Some participants of the meeting felt that failure to openly challenge structural and social injustice and violence is to tolerate or be complicit in attempts by others to limit rights for sexual and gender minorities, while others felt that quiet efforts with key allies and opponents carries lesser risks in terms of exacerbating negative attention and social responses, and has greater potential to make a lasting positive difference in the longer term. In the amfAR guidance it is noted that “*a delicate balance* [has to be struck]” [27]. They note that “*The research agenda should not supersede the community’s interest, and developing strong partnerships with MSM/LGBT individuals and community organisations may reduce the likelihood of researchers ‘getting out in front’ of the community on rights issues*” ([27] p. 9). The suggestion in this quote is that allowing researchers, instead of community representatives, to take a lead role in advocacy has the potential to backfire and have negative effects on MSM communities that have not yet decided upon that particular approach to tackle their circumstances.

Over the course of our meeting, it was clear that the different emphases in approaches to advocacy can seem to be in conflict: an engagement approach that emphasises health provision for all can appear to fall short of, and even undermine (through focusing energies on such initiatives), a deeper and potentially more challenging goal that tries to overthrow discriminatory laws against homosexuality. For public health researchers and engagement researchers and practitioners in health projects, a public health approach (where the focus is on health and rights to health) can feel a more appropriate entry point to openly challenge underlying social and structural violence and inequities. However, wider agendas were also suggested for researchers that included advocating rights to sexuality, questioning the criminalisation of homosexuality, and empowerment of MSM in other social issues. It was recognised that a particular challenge or opportunity for research is in national laws and policies, with some countries demonstrating conflicting positions on MSM rights (for example, rights to health for all versus outlawing of homosexuality). Overall, it appeared to be recognised that different individuals may take on different types of advocacy roles in different contexts and at different times, and that multiple approaches in a given programme of work or network might be appropriate and valuable. There was consensus that, in any context, developing appropriate links, collaborations and networks of individuals and groups with different strengths, emphases, and ways of working is likely to be essential.

A third area of difference and debate was in thinking about the role of engagement in broader discussions of the ethics of research and study design. Some participants appeared to see CE with MSM as a critical part of the science and ethics of a research endeavour, but not the entire endeavour. For these participants, CE in much health research (with the exception of participatory research) involves the interactions, discussions and mutual learning that occurs between researchers (who may or may not be open members of MSM communities themselves) and representatives of a wide range of different communities, including principally, but not exclusively, representatives of MSM. All of these interactions and discussions inform research design and implementation strategies, including specific ethical issues such as benefits and risks during and after research. However there are also other important inputs into research design, including health, research and ethics expertise which may be contributed from non-MSM communities. For other meeting participants, MSM studies appeared to be seen as all about, or taking as the starting point, engagement with MSM communities. The implication of the latter position is again encapsulated by the phrase ‘nothing about us without us’; suggesting that all research involving MSM should be led in design and implementation by MSM communities as is standard in participatory research designs. For those who saw CE with MSM as critical but not the entire research endeavour, there was a concern that critical aspects of ethical research, such as careful consideration of potential study risks, individual autonomy, and what responsibilities researchers have for study participants, have the potential to be lost in debates about which MSM communities to involve and who appropriately represents different MSM communities, as discussed in more detail below.

Overall, across all of the above three areas, we agreed at the meeting that any research involving MSM should recognise that some health needs of MSM are shaped by and connected to deep rooted structural injustice, and that researchers should endeavour to engage with these issues in locally appropriate ways as discussed further below. Exactly how this is done requires careful discussion with relevant local communities and other stakeholders.

## Developing community engagement plans

When planning CE in MSM research, we suggest several general dimensions to consider, before moving onto specific examples and experiences from our work of CE with MSM (summarised in Table 1) and other communities, or the broader public (Table 2).

**Table 1:** Examples of goals, successes and challenges in engaging with MSM communities

Examples of goals of engagement	Suggestions based on field experience	Examples of issues and challenges
Research		
• Better designed and implemented studies (questions, ways of working, benefits, CE)	• Include diverse representatives of key populations in a range of interactions in designing studies, how they are implemented and feedback • Community representative groups such as CABs/Gs may be considered as a key channel of engagement • Ensure all research team members and CE partners understand the research and CE goals, limits and messages	• If/how to engage those who choose not to be identified? Risk failing to engage with those who do not feel represented by self-proclaimed ‘representatives’ • Potential to breach confidentiality and exacerbating risk in interactions with representatives • Should CAB/Gs include non-MSM representatives? If not, should separate CAB/Gs be established to engage with other community members? (Table 2)
Public health		
• Improved health and well-being for MSM • Improved health and well-being for general communities	As above and • Provide evidence to support MSM-friendly policy change • Providing MSM-specific access to healthcare with MSM friendly staff • Where possible, provide services to a wider range of community members than currently access services, and do not make access to such services dependent on research participation • Lobby (through networks) for health policy change to support improved health and well-being for MSM as important part of general community • Link with LGBT organisations to refer to services beyond medical research and care	As above and • Key messages about health research involving MSM not carefully worded and understood by all research team members can be stigmatising for MSM • Activities can contribute to MSM discrimination by targeting MSM communities as specific beneficiaries of interventions that many others would appreciate; such activities also separate and make more visible MSM from other community members • Activities may be interpreted as promotion of homosexuality, exacerbating MSM discrimination
Human rights/social justice		
• Advocating for legal changes • Empowerment of MSM individuals and communities	As above and • Lobby for specific health and social rights of MSM • Hire LGBT staff • Provide training about LGBT issues to all staff and healthcare providers	As above and • Failure to act may result in tokenistic research agendas and contribute to structural violence • May need to operate covertly to ensure safety of participants and operations

CAB/G, Community advisory boards/groups; CE, Community engagement; GMT, Gay-Other MSM-Transgender; LGBT, Lesbian, gay, bisexual, and transgender; MSM, Men who have sex with men.

**Table 2:** Examples of goals, successes and challenges in engaging with broader communities for studies involving MSM communities

Examples of goals of engagement	Successes and suggestions	Examples of issues and challenges
Research		
• Researchers better understand the social context of the research and have the potential to hear about and respond to issues before they become serious problems	• In communications about research, one meeting participant described it as helpful to explain their interest in health research broadly – not just for example among MSM	• Communications aimed at explaining research or support for MSM can be understood, interpreted or otherwise shared in the broader community as promotion of homosexuality
Public health		
• Increased awareness in local communities that MSM exist and that some have serious health and broader vulnerabilities	• Several meeting participants discussed the value of focusing CE discussions on behaviour (for instance anal sex) regardless of sexuality to minimise ‘othering’, or the perception that that health concerns do not apply to heterosexuals	• Tendency especially in public meetings for simplification of issues, or issues being sensationalised; e.g. – there can be a conflation of identity with risk practice – risk information can be translated or interpreted as MSM communities being ‘dangerous’ to the broader community as opposed to vulnerable to some health problems
Human rights/social justice		
• Changed attitudes towards MSM in communities where research is being conducted	• The general community involves many overlapping sub-communities based on, for example, business interests, gender and religion; workshop participants described the importance of regular and often informal interactions with as many communities as possible	• Language and otherness – ‘they’ falsely distinguishes MSM from the broader or ‘general’ community

CE, Community engagement; MSM, Men who have sex with men.

### General considerations in planning CE for MSM research

The broader CE literature suggests that researchers need to carefully consider which communities to engage with and that this is likely to include people beyond the MSM community themselves, including local community chiefs and elders, the general public, health workers/managers, media, and LGBT/GMT groups [1]. For each community to be engaged, the goals, strategies, activities, indicators of success and potential challenges might differ. We recognise that decisions regarding communities and activities will necessarily depend on the sociocultural and political context, the study aims and approach, and the nature of the institution(s) leading or driving the research. Furthermore, there will be economic considerations, including what is feasible to pay for under CE (such as travel expenses, mobilisation activities, get-togethers, radio programmes, science fairs). Also any payments made, for example, to support recruitment and retention of participants, need very careful ethical consideration given the potential for under-reimbursement or, conversely, undue inducement, for example [30, 31]. Specific considerations include:

- It can be helpful in the planning of CE for MSM studies to define CE as communication and interaction about research and associated activities (for example, discussions about what kinds of health services should be provided to participants), rather than as the actions or ways of working that are identified or agreed upon through that process of participation (for example, the health services that are provided as a result of CE discussions). In so doing, however, we note that these engagements will discuss critical ethical issues including designs of studies, benefit sharing approaches, recruitment and confidentiality procedures, and advocacy strategies.
- Defining communities to engage with is a complex and ultimately contrived activity because people are members of multiple communities, membership of communities changes over time, and communities can be defined differently by different people such as researchers and ‘community’ members themselves [32]. Defining MSM communities specifically, and selecting appropriate, widely agreed ‘representatives’ of MSM communities, is particularly complex in contexts of stigma and segregation, with many MSM not self-identifying as MSM, and not necessarily participating in any MSM communities, however defined.

In considering the goals of engagement, it may be helpful in CE plans to carefully consider the multiple intentions of interactions with specific communities, possibly ranging from communication (simple information sharing), through consultation (seeking advice and selecting whether or not to act upon that advice depending on a range of considerations) through to partnership. A crucial question is what if the varying goals for engagement are in conflict with each other? For example, the goals of respecting social and cultural attitudes on homosexuality may, in some contexts, be seen to be in conflict with respectful engagement of MSM in HIV vaccine trials.

In recognition of some of the above complexities, we agreed at the meeting that CE goals and activities ideally, at least with MSM communities but also with other publics, can and should be identified at the early planning stages of research. Preferably, this process would occur prior to sourcing funds and science/ethical review, in order to allow for changes to study design and implementation, many of which may have important funding implications. Furthermore, regardless of how early engagement starts, is it essential that plans constantly shift over time in response to unfolding realities. As complex social interventions in changing and often hostile environments, studies and associated engagement activities inevitably have to change over time. While check lists are helpful in CE, action should not be reduced to a tick box type activity failing to look out for and seek to alleviate negative outcomes. Falling into a tick box type approach to CE risks undermining the fundamental aim of much CE, which is to strive to ensure that research is safe and beneficial for MSM individuals and communities.

### Experiences and challenges with engaging specifically with MSM communities in research (Table **1**)

Given that a key ethical concern for MSM research is to ensure that research is safe and beneficial for MSM individuals and communities, researchers need to centrally engage with members of MSM communities and LGBT/GMT groups throughout studies, including study conception, design, implementation and dissemination of findings. There are often limitations to researchers’ capacity to do this meaningfully, in terms of knowledge and skills, but it is essential to understanding the potential benefits and unintended adverse outcomes that might occur as a result of research. Members of MSM communities can advise upon appropriate language, definitions of community, and advocacy approaches for a particular study or set of studies, in a given socioeconomic, political and institutional context, and upon protection and support should adverse outcomes emerge.

As noted above, a challenge is that even identifying and working with different MSM sub-groups risks inappropriate labelling and the creation of false categories that do not resonate with reality or that benefit some at the expense of others, and possibly raising stigma and discrimination. Further ethical needs and challenges discussed in the meeting with regards to engaging with MSM representatives were:

- Honest and open communication about what the goals of engagement are and what can be achieved for MSM communities through research, given what are often critical and multi-faceted needs;
- Ensuring that what is promised and given to MSM participants and communities as part of research is not organised in such a way as to undermine individuals’ abilities to make free and informed choices about whether or not to participate in that research;
- Avoiding being drawn into internal conflicts within and between different LGBT/GMT groups in such a way as to undermine research benefits or increase vulnerability of MSM participants or communities; and
- Seeking out and taking into account the views and priorities of arguably the most vulnerable MSM – those who chose not to be identified and who do not necessarily identify with GMT – while ensuring that those individuals’ identities are not exposed to others against their will.

A fundamental ethical challenge in engaging with MSM representatives highlighted at the meeting was regarding representation; who is representing whom, in what way, and with what intention and outcome. Discussions also illustrated that, although creating democratic, safe spaces may be important for MSM communities, there are also risks associated with doing this, such as increasing visibility of individuals and the communities they belong to, and raising concerns, jealousies and negative action by others in the community that in a homophobic environment may lead to violence. Such negative perverse outcomes have the potential to undermine the fundamental value of research and the activities aimed at supporting MSM communities on a day-to-day basis and for long-term structural change.

### Experiences and challenges of engaging with other communities or publics (Table **2**)

There are specific concerns regarding engaging other communities over the course of planning, implementation and completion of studies where MSM are research participants. These groups could include the general community of a geographical location including the family members of the MSM participants, religious leaders, health workers, the media, policymakers and advocacy groups. On the basis of the experiences of workshop participants, there are several over-arching goals for communicating with such communities (see Table 2 to illustrate goals, challenges and successes of engaging with general communities). One goal is to promote awareness among these communities that MSM exist despite their invisibility, that they have serious health and broader vulnerabilities, and that they have a right to health (and safety) as with the rest of the population. A second overarching goal is to enable researchers to understand the sociocultural and political context in which the research is being conducted, and how the research is being reacted to. A third is to try to change attitudes to MSM in these communities.

There are a range of ethical challenges in engaging with these diverse communities; as well as strategies to, counter strategies to mitigate these challenges:

- Explaining to the general population the public health reasoning for working with MSM (for instance, explaining that MSM are one of the key populations in Kenya at higher risk for HIV infection, and that together with, for instance, female sex workers, and injecting drug users, they are actively mobilised by national programmes to benefit from HIV prevention and antiretroviral therapy care programming) can be misinterpreted as promotion of homosexuality which is highly sensitive in many homophobic contexts in sub-Saharan Africa;
- The tendency in many communities for issues to be sensationalised, and more specifically for information about risk or vulnerability to be translated as MSM communities being ‘dangerous’ or a threat. Thus, for example, information about relatively high levels of HIV among MSM populations may lead to stereotypes that being MSM is associated with being HIV positive, and that MSM are guilty of driving the spread of HIV in heterosexual populations;
- Linked to the previous point, references in interactions to MSM as ‘them’ or ‘others’, positions MSM as being other than, and apart from, the general community, which can feed into segregation and discrimination against MSM;
- The introduction of secondary stigma to those who are working with and supporting care for MSM individuals and communities.

The above messaging may be valuable in reducing stigma, and can be uniting when communicating with MSM communities to explain why other key populations are also eligible for some research-related activities and benefits. However, these two sets of messaging can sometimes conflict: information about relatively high HIV levels among MSM can increase the stigma and isolation that these populations face. Such challenges are not easily resolved and illustrate the complexity of CE initiatives in many contexts.

These points highlight the ethical dilemmas associated with engaging with different individuals and communities who do not necessarily share the same views and values. Although everybody has a right to hold their own values, a challenge for MSM research is that some values are discriminatory and others remain unheard. It is essential that key messages to these groups are carefully worded to minimise negative stereotyping and misconceptions that reinforce stigma.

## Researchers’ responsibilities in research and engagement

CE is an important way of gauging ethics in context and making research meaningful for various publics; it is important for ethical research and for conducting socially responsible science. However, we have shown that it can also contribute to a range of harms. Researchers should be aware of potential harms, and should not think of CE as a one-stop solution – it is essential to take other research ethical issues into consideration. Here, we summarise the key insights of the meeting for the responsibilities of researchers and, in what follows, offer some suggestions for further research and highlight the lessons learned.

The structural violence connected to health needs of MSM in sub-Saharan Africa [13] is an important driver of research in the region, but also presents ethical challenges and potential harms for both research and CE practice. As noted above, harm can be caused through labelling, breaches in confidentiality, increased visibility and stigma, and threats to safety. These harms can be inflicted on individuals, their wider communities, or those involved with or otherwise supporting MSM, including family members, health providers, advocacy groups and researchers. Another potential harm in MSM research is a lack of transparency by researchers (intended or not) about the goals of research, and about the limits of what research can provide and achieve in the context of multiple needs. A disjoint in the goals for research and engagement between different participants (e.g. between responding to more fundamental needs and health outcomes) can lead to failure of trust.

In the literature there has been growing emphasis on the importance of researchers recognising macro-level or structural ethical issues for participants (such as poverty and lack of access to healthcare) as well as micro-level or individual issues (such as ensuring voluntary informed consent and balancing risks and benefits of participation in particular studies) [33–38]. It is recognised that researchers’ potential to address macro-level structural inequalities may be more limited than taking on micro-level responsibilities, but that one substantial way to tackle macro-level responsibilities is through supporting or providing initiatives that benefit all community members, regardless of participation in research activities (i.e. ‘community-wide benefits’, for instance, a population-wide STI and HIV clinic) [37–40], in this case, for example, improved access to appropriately run medical services for as many members of MSM communities as possible [41, 42].

In our discussions, and drawing on existing literature, it was felt that, as health researchers, responsibilities should begin with meeting the needs and responsibilities that are closest to the research goals, such as reduced HIV transmission as opposed to homelessness or access to employment. At a minimum, for participants, researchers have to ensure that research risks and any collateral damage by research are identified early, and that there are plans (or mechanisms) in place to ensure appropriate ancillary care, referrals for support or other means of mitigating harm. This requires the perspectives and concerns of the target population to be heard. To ensure that voluntariness is supported, and the potential for stigma is minimised, this support may need to be offered beyond participants and beyond MSM communities, although the latter decision has to be counter-balanced against the need for safe spaces, which might be compromised with shared activities and interventions.

The above approach begins to meet both macro- and micro-level responsibilities of researchers, in ensuring that participants still have some choice about whether or not to participate in research, and that benefits reach a broad community. However, this relatively minimal approach does not tackle the underlying causes of many social and health needs, and risks researchers being complicit in sustaining those structural drivers. Given the time-bound limitations of many research projects, what are often essential (sometimes implicit) ties with governments, and the inability of most biomedical researchers to act as development practitioners, the sense in the meeting was that to tackle structural injustices, biomedical research projects will often have the responsibility to build relationships and agreements not only with government, but also with other institutions and networks with the expertise and experience to engage with structural issues, including, for example, health organisations, LGBT/GMT groups and other health and human rights advocacy groups. Such institutions and networks may have a longer presence in the community and potentially greater impact on policy and practice than researchers. Researchers can learn about research needs from these institutions and networks, and researchers can provide them with potentially useful data and lessons to make a positive change.

Having noted the importance of developing appropriate collaborations, we recognise that MSM research is an inherently socially and politically charged activity. Researchers are therefore likely to have to consider where on the advocacy spectrum they place themselves (from open advocates to offering what we have called the ‘under the radar’ strategic inputs) and to do this based on a good understanding of the sociocultural, political and organisational contexts in which they work. This positioning is likely to require careful reflection of what actions and approaches are most likely to benefit MSM communities and minimise harms, linked in part to researchers’ own ability to communicate about potentially highly sensitive topics with different stakeholders in particular contexts. Decisions will require researchers to be reflexive on factors influencing their stance and potential influence, including their own expertise, experience, networks, identity and ethnicity, their institution’s role and reputation locally and nationally, and the shifting opportunities and challenges for policy impact during and after research [43–45].

Regardless of where we as researchers place ourselves on an advocacy spectrum, we recognise that in our day-to-day practice, there is a potential to continuously build up our ‘ethical mindfulness’. Guillemin and Gillan [46] argue that researchers can do this through (1) acknowledging the role of ethically important moments in the everyday practice of research; (2) giving credence to ‘not feeling quite right’ about a research situation; (3) articulating what is ethically important in the practice of research through application of the principles of respect, justice and beneficence; (4) being reflexive, that is, taking stock of actions and their role in research; and (5) having courage by way of being receptive to new ways of thinking about research ethics and critically challenging established research practice. These suggestions are invaluable to us all in our continued work. Working to include communities in ethical reviews and reflections could help reduce the possibility of formal guidelines and approaches, including for CE, being reduced to routinized activities that lose sight of the goal of ensuring that research is safe and beneficial for MSM individuals and communities.

## Towards a research agenda

In the meeting and in this paper, our intention was to raise and debate complex issues arising from the field, rather than to provide firm, normative answers. This activity was informed by recognition that CE discourse and practice for research among MSM in sub-Saharan Africa had the potential to differ significantly from experiences in other parts of the world. As is the case for CE more broadly [1], there are many areas where further reflection and research are needed, including through innovative methodological approaches such as participatory, deliberative, reflexive and philosophical approaches. Given the ethical nature of many of the issues and dilemmas raised, empirical ethics studies are likely to be particularly valuable, where empirical data are combined with ethical analysis to allow normative claims [47]. Similarly, given the need often for an in-depth understanding of unfolding and sensitive realities on the ground, in particular for diverse members of MSM communities, detailed anthropological and political studies, including by or with members of MSM communities, are likely to be essential.

Potential topics for follow-up through empirical research include:

- Who represents MSM in research engagement activities, and what are their links and relationships with diverse MSM communities? Research could include examining who is selected or puts themselves forwards as representatives, if and how they link with those they represent, and different stakeholders’ perceptions of strengths and challenges of working with these representatives. This work in particular would be strengthened by in-depth approaches aimed at understanding the range of MSM communities in research settings, including those who are least vocal and visible, and their priorities and concerns with regards to research, practice and representation.
- What does CE mean and how does it ‘work’ for diverse MSM and other communities across different contexts? This work could explore the potentially diverse goals that different actors involved in CE activities might have, if and how these goals are met and shift over time, and with what consequences (both positive and negative) for different individuals, their communities and research. This research could include examinations of how key principles for research involving MSM are developed and shared with different stakeholders in ways that are open, honest and safe, and if and how power relations between researchers and community members are shifted. The findings would strengthen researchers’ understanding of CE realities, and inform future CE strategies and processes.
- How can researchers and funders define their responsibilities towards research participants and the communities they are part of in varying contexts? What are researchers providing to participants and their communities for different kinds of studies involving MSM, on what basis, and what are different actors’ perceptions about the appropriateness of these actions and provisions? What does ethical analysis teach us about what researchers should provide, and the basis for these responsibilities? This should include consideration of how the following should be balanced: compensation of real costs and time; appreciation of research contribution; respectful engagement with participants; avoidance of undue inducement; potential to introduced unfairness and relationship problems between research participants and others; and the interest to transform structural drivers of inequity and stigma.
- How can health researchers’ engagements with diverse communities contribute to tackling the underlying structural factors that contribute to health problems, stigma and discrimination? Documentation of existing approaches by researchers themselves, and across their networks and collaborators, and strengths and challenges encountered could contribute to literature and ideas around research-policy-practice (‘research uptake’ or ‘knowledge transfer’) approaches for studies involving MSM. Policy analysis, including examination of what contributed to policy change and how, is likely to show if and how research findings and other advocacy efforts feed into change, and could suggest strategies for researchers and their collaborators.

## Conclusion and lessons learned

### Cross cutting issues in community engagement approaches

- The language used in conducting studies with MSM is ethically charged, and can have important implications for what CE takes place, and how it is understood and responded to by members of diverse communities.
- A broadly public health- or rights-based approach to CE may lead to differences in perspectives on appropriate forms of CE, with the former focusing on general expectations of socially responsible researchers in contexts of major shortages and inequities, and the latter on the very specific vulnerabilities and challenges faced by MSM and having a more politicised approach.
- Important points of convergence across the two approaches to CE include recognition that both approaches include a mix of often poorly articulated instrumental and intrinsic goals for CE; different individuals may take on different types of advocacy roles in different contexts and at different times, including through collaborations and networks; and debates about defining MSM communities and their representatives should not overshadow other critical aspects of ethical research, such as careful consideration of potential study risks, individual autonomy, and what responsibilities researchers have for study participants.

### Developing CE strategies and being alert to potential harms

- Studies involving MSM need to carefully consider which communities to engage with, and what the different goals, activities, indicators of success and potential challenges might be for each. Given the complexities and sensitivities of defining communities and representatives, planning of these activities should begin as early as possible, and respond to unfolding realities. Specifically, researchers need to build capacity to engage appropriately with LGBT/GMT groups and other members of MSM communities, and with religious leaders, health workers, the media, policymakers, and advocacy groups.
- Ethical issues in CE include who represents diverse MSM needs and realities, and particularly the priorities and concerns of those who are least vocal and visible, and the potential to cause harm through labelling, breaches in confidentiality, increased visibility and stigma, and threats to safety. Further potential harms include a lack of transparency by researchers about the goals of research, and limits of what research can provide and achieve.

### Researchers’ responsibilities and the limits of these responsibilities

- At a minimum, researchers have to ensure that research risks and disadvantages for participants are identified, and that there are mechanisms in place to ensure appropriate ancillary care, referrals for support or means of mitigating any harms identified.
- The health needs of MSM are often shaped by and connected to deep rooted structural injustice. Researchers have a responsibility to engage with these issues in locally appropriate ways, including through developing a good understanding of the contexts in which they work, and building appropriate links, collaborations and networks with those with appropriate knowledge and skills about how to make a positive change in policy and practice.
- Researchers conducting studies involving MSM may benefit from research ethics training, including a relevant (targeted) ethics curriculum. However, more broadly, it is vital that researchers build ethics support, advice and research into studies involving MSM from the earliest stage and throughout and after studies, to ensure that ethical challenges are identified and engaged with as and when they arise.
